# Small-statured tomato plants for automating greenhouse production: opportunities, constraints, and research priorities

**DOI:** 10.3389/fpls.2026.1966159

**Published:** 2026-10-28

**Authors:** Zixin Yao, Leo F. M. Marcelis, Ad N.M. de Koning, Gene A. Giacomelli, Tadahisa Higashide, Ep Heuvelink

**Affiliations:** 1 Horticulture and Product Physiology, Department of Plant Sciences, Wageningen University & Research, Wageningen, Netherlands; 2 Ridder, Maasdijk, Netherlands; 3 Controlled Environment Agriculture Center, The University of Arizona, Tucson, AZ, United States; 4 National Agriculture and Food Research Organization, Institute of Vegetable and Floriculture Science, Tsukuba, Japan

**Keywords:** autonomous production, dwarf tomato, high-wire tomato, labour requirement, light use efficiency, limited-truss tomato, small-statured tomato

## Abstract

Systems based on small-statured plants could contribute to the future of automated tomato production, but require an integrated approach of cultivation, crop management, plant physiology, breeding, robotics, and modelling. Greenhouse tomatoes are typically grown according to a high-wire system, where plant stems reach 10 m or more in length during one cycle of production. This system has served the industry for about 50 years, and allows for near-continuous year-round production, high yield, and good fruit quality. However, it is labor intensive, requiring manual fruit harvest, pruning of side shoots and leaves, as well as training the stem around the support string. The viability of high-wire cultivation is now challenged by high labor costs and shortage of workers. In this perspective paper, we propose two alternative tomato production systems: dwarf tomato and limited-truss tomato (LTT). They both produce small-statured tomato plants with short cultivation cycles, which may improve compatibility with automated production. We evaluate and compare the two production systems based on yield, light use efficiency, fruit quality, and labor requirement. Future directions for both research and industry for innovative tomato production are presented as well.

## Introduction

Tomato is one of the most popular vegetables worldwide. From 2003 to 2023, the global tomato production has rapidly increased from 118 Mt to 192 Mt (FAOSTAT, n.d). Currently, the whole-season greenhouse tomato production is based on the high-wire system, where stems are trained to grow along plastic twine hung from overhead horizontal wires. With the periodic removal of lower leaves and fruit harvest, the stems are continuously lowered and moved along the wire.

Typically, in the high-wire system, the plant height may exceed 3 m depending on the greenhouse height; and the stem length could be more than 10 m, producing about 35 trusses during an 11-month growing season. A large amount of labor is required for weekly crop management practices that include training, pruning, and harvesting. Labor represents on average 31% of total costs in the Netherlands (Raaphorst et al., 2026). Contrary to increasing labor costs and reduction in labor availability, the labor hours required per hectare of greenhouse has remained unchanged over the last fifteen years (van Horen, 2025). Although robots have been developed such as commercially available old-leaf removal robots (Priva, 2021; Tomatoworld Foundation, n.d) and experimentally demonstrated fruit-harvesting robots (Matache et al., 2026), the implementation of automation in high-wire systems remains challenging due to the tall and continuously changing canopy. Thus, an alternative production system that reduces manual labor and facilitates robotization is urgently needed. One possibility of automation in a high-wire system is developed by SAIA, where tomato plants are moved to robots for scanning, harvesting and leaf pruning, supported by AI-based plant monitoring (Bentvelsen, 2025).

One of the alternatives is to grow dwarf tomato cultivars with a determinate growth habit. Determinate growth is primarily related to a recessive mutation in the SELF-PRUNING (SP) gene (Carmel-Goren et al., 2003), whereas dwarf stature may involve separate genes affecting internode elongation, such as the DWARF mutation reported in Micro-Tom (Martí et al., 2006). They are also called micro dwarf, super dwarf, or compact tomatoes and are different from dwarf tomatoes grown in open field. These names reflect their highly compact architecture (plant height under 40 cm). They have a short life cycle (70–100 days) (Ke et al., 2022) and concentrated fruit-setting period, which is advantageous for mechanical harvesting (Carmel-Goren et al., 2003) and are suited for vertical farming.

Another alternative is to grow indeterminate genotypes with only one or a few trusses, by pinching the growing point of the plant above the first or first few trusses. Giacomelli et al. (1994) referred to this system as the single-truss tomato production system, while Takahashi et al. (2012) described it as a low node-order pinching system at high plant density. Here we refer to it as the Limited-Truss Tomato (LTT) production system. Compared to the conventional high-wire system with many trusses simultaneously at different developmental stages from anthesis to harvestable fruits on a plant, the LTT has a simpler plant morphology with compact plants and fruit trusses at similar growth stages, allowing for automation and robotic handling and harvest as well as vertical farming.

## High-wire system

*Yield* – Unlike determinate or LTT systems, high-wire cultivation allows for an extended production cycle, often lasting about 11 months, with fruit production continuing for approximately 9 months. Plants are typically grown with a planting density of 2.5 stems m^-2^, and during the summer season, some side shoots are retained, resulting in about 4 stems m^-2^ (Heuvelink, 2018). In the past 40 years, the annual yield of large-fruited high-wire tomato production has significantly increased from 30 to 72 kg m^-2^ in the Netherlands, and with supplemental lights, which are typically Light-Emitting Diodes (LED) nowadays, this can be as high as 111 kg m^-2^ (**Table 1**, Heuvelink et al., 2025; Raaphorst et al., 2026). These large yields result from both high biomass production and high harvest index (ratio of fruit dry weight to total above-ground plant dry weight), which is about 0.7 (de Koning, 1994). However, the annual yield of cherry tomato was much lower, at 34 kg m^-2^ with supplemental lighting in the Netherlands (Raaphorst et al., 2026).

**Table 1:** Overview of several aspects of high-wire, dwarf, and limited-truss tomato (LTT) production systems.

	Production system				
Properties	High-wire			Dwarf	Limited-truss
	Large-fruited	Cherry			
Planting density	2.5-4 (stems m^-2^)			20-100 (plants m^-2^)	10-14 (plants m^-2^)
Fruit size (g per fruit)	150-190		Not reported	7-9^a^	170-210^a^
Yield per crop cycle (kg m^-2^ per cycle); with supplementary light	111		34	6-9	6-15^b^
Cultivation cycle	11 months			70–100 days	~100 days
Annual Yield (kg m^-2^); with supplementary light	111	34		30–45	24-60^b^
LUE (g fruit FW^c^ mol^-1^ incident photons)	12^a^	4^a^		7-11	3-5^a^
LUE (g fruit DW mol^-^¹ incident photons)	0.5^a^	0.4^a^		0.7-1.1^a^	Not reported
Harvest index	0.7			0.5–0.6	0.4-0.6
Cultivation-related labor requirement (h ha^-1^)	7,500	17,000		4,000^a^	5,400-5,900^b^
Cultivation-related labor requirement (min kg^-1^)	0.4	3.0		0.5-0.8^a^	0.54-0.59^b^
Soluble solids (°Bx)	4-6	6-14		5-7, dynamic planting density can improve Brix	Similar to high-wire, but can achieve higher Brix by imposing End-of-Production salinity stress

Literature sources for numbers presented are in **Supplementary Table 1**.

Values without superscripts are directly reported in the original sources. ^a^Calculated or derived from data reported in the original source. ^b^Estimated from data reported in the original source using additional assumed values or parameters. ^c^FW, fresh weight; DW, dry weight.

*Light use efficiency* – One of the key factors contributing to the increase in yield in high-wire systems by breeding over the period 1950–2000 is the improvement in light use efficiency (LUE) (Higashide and Heuvelink, 2009). In this study, LUE is expressed as grams of fruit FW or DW per mole of cumulative incident photons and was calculated using annual fruit yield and radiation and supplementary lighting data from KWIN (Raaphorst et al., 2026). Incident PAR was estimated from outdoor radiation, assuming 70% greenhouse transmissivity (Kempkes et al., 2020). Therefore, the estimated LUE was 12 g fruit FW mol^-^¹ incident photons for large-fruited tomato and 4 g fruit FW mol^-^¹ incident photons for cherry tomato. With dry matter contents of representative modern cultivars grown in greenhouse (Hemming et al., 2020; Kusuma et al., 2023), the DW-based LUE were approximately 0.5 and 0.4 g fruit DW mol^-^¹ for large-fruited and cherry tomatoes respectively. The smaller difference in DW-based LUE is due to the higher dry matter content of cherry tomato. Moreover, environmental factors such as carbon dioxide concentration, air temperature, and relative humidity also influence LUE (Cocetta et al., 2017; Huber et al., 2021; Verheul et al., 2022).

*Fruit quality (Brix)* – Tomato fruit quality in high-wire systems shows considerable variation. Quality parameters such as total soluble solids (°Bx), total titratable acidity, and fruit firmness, all indicators of taste, differ substantially among cultivars and fruit types. For example, total soluble solids of large-fruited tomatoes generally range from 4 to 6°Bx (Quinet et al., 2019). Cherry tomatoes usually have higher Brix values, ranging from 6 to 9°Bx (Bhandari et al., 2016), while some high-sugar cultivars can reach up to 14°Bx (Ino et al., 2023).

*Labor requirement* – Labor requirement in high-wire tomato production is high due to the intensive crop management practices and the long cultivation cycle. Labor tasks in this system can be categorized as: (1) preparations, including planting and the setup of gutters and irrigation systems; (2) crop management, including training, leaning, clipping or wrapping the plant around the support twine, leaf and side shoot removal, and fruit pruning; (3) pest control; (4) harvesting; and (5) cleanup. In Japan, the average annual labor input in high-wire tomato was 11,293 h ha^-1^, with the majority of time spent on tasks performed on a raising platform, such as lowering, leaning, and clipping of plants (35%), followed by harvesting (31%), and leaf removal (15%) (Kurosaki et al., 2014). In the Netherlands, annual labor requirement for high-wire cultivation of large-fruited tomatoes is approximately 7,500 h ha^-1^ and 0.4 min kg^-1^, but these numbers can reach up to 17,000 h ha^-1^ and 3.0 min kg^-1^ for cherry tomato (Raaphorst et al., 2026).

## Dwarf tomato

*Yield* – The short growth cycle and compact plant architecture of dwarf tomato enable multiple crop cycles per year and dynamic planting density, in which planting density is gradually decreased during the cultivation. In the Autonomous Greenhouse Challenge, with supplemental lighting, annualized yields of dwarf tomato were estimated at 30–45 kg m^-2^, depending on the cultivation strategy, including dynamic plant spacing and an average planting density of around 30 plants m^-2^ (Maree et al., 2025). Reported harvest index of dwarf tomato is lower than that of high-wire, and ranges between 0.5-0.6 (Karpe et al., 2024).

*Light use efficiency* – The LUE of dwarf tomato varies between 7–11 g fruit FW mol^-1^ incident photons (Maree et al., 2025) and 0.7-1.1 g fruit DW mol^-1^ incident photons assuming 10% fruit dry matter content (Karpe et al., 2024). When LUE is calculated based on incident light, it is strongly affected by planting density. Increasing planting density enhances light interception of the canopy and therefore increases LUE based on incident light (Karpe et al., 2024). However, light capture usually reaches a maximum value at a leaf area index (LAI) of 3 to 4 (Heuvelink et al., 2005).

*Fruit quality (Brix)* – Reported Brix values of dwarf tomatoes range from 5 to 7°Bx, depending on cultivar and growing conditions. For example, ‘Micro-Tom’ reached 5.3–5.9°Bx under different nutrition rates, while cultivars such as ‘Micro Tom’, ‘Jochalos’, and ‘Venus’ exhibited cultivar-dependent Brix values of 4.9–5.5°Bx (Terada et al., 2023; Tharpe, 2023). In addition, fruit quality is also affected by planting density. For ‘Plum Tomato Red’, Brix decreased from 9.4°Bx at constant low-density (19 plants m^-2^) to 6.6°Bx at constant high-density (100 plants m^-2^), whereas dynamic plant spacing maintained values of 8.9 – 9.4°Bx (Karpe et al., 2024). Employing dynamic plant spacing can mitigate the reduction in fruit quality associated with high planting density, while achieving a high yield compared to low planting density.

*Labor requirement* – Unlike high-wire systems, dwarf tomato production does not require labor-intensive tasks such as lowering, leaning, clipping, training, and leaf removal. Estimating labor requirements for dwarf tomato cultivation is challenging because of limited data availability. To address this issue, the labor requirement of *Bromelia Vriesea* grown on mobile benches was used as a proxy, as it has a comparable pot size (12 cm) and planting density (33 plants m^-^²) to the dwarf tomato system, and also uses supplemental lighting (Maree et al., 2025; Raaphorst et al., 2026). The reported labor requirement is 4,000 h ha^-^¹ yr^-^¹ (Raaphorst et al., 2026), corresponding to approximately 0.5-0.8 min kg^-1^ using an annualized yield of 30–45 kg m^-2^. On an area basis, this is lower than both large-fruited tomatoes (150–190 g, 7,500 h ha^-1^) and cherry tomatoes (17,000 h ha^-1^) in high-wire system in the Netherlands (Raaphorst et al., 2026).

## Limited-truss tomato

*Yield* – Theoretically, many tomato cultivars suitable for high-wire cultivation can also be grown in LTT systems. Reported yields in LTT systems typically range from 3 to 15 kg m^-2^ in one growth cycle, depending on fruit type, planting density, the number of trusses, and environmental conditions such as supplemental light (Jiang et al., 2017; Lu et al., 2012b, a; Tewolde et al., 2016). The reported maximum planting density in LTT is 14.3 plants m^-2^, which partially compensates the low individual plant yield (Jiang et al., 2017). With a growth cycle of three months, excluding the four weeks of seedling production after sowing, four rounds can be achieved per year, resulting in an estimated maximum annual yield of 60 kg m^-2^. Similar to dwarf tomato, reported harvest index of LTT ranges from 0.4 to 0.6, which is lower than that in high-wire tomato (Jiang et al., 2017; Sato et al., 1994).

*Light use efficiency* – Generally, LUE of LTT is lower than that in high-wire, as the harvest index is low due to the low number of fruits. However, directly comparable LUE data for LTT is limited. A LUE of 3–5 g fruit FW mol^-1^ has been calculated from data of Itoh et al. (2020) based on incident photosynthetically Active Radiation (PAR) when three trusses were kept.

*Fruit quality (Brix)* – Given that cultivars grown in high-wire can also be grown in LTT, cultivar-related differences in fruit quality are likely rather small between the two systems. However, in LTT, there are possibilities to improve fruit quality that cannot be applied in high-wire. For example, a high Brix value can be achieved by imposing salinity stress during fruit ripening stage, without adverse effects on later trusses or further vegetative growth (Sakamoto et al., 1999). Compared with a constant EC of 2, an EC of 8 dS m^-1^ applied from fruit turning stage in LTT increased Brix from 6.1 to 7.1 without a significant reduction in yield (Sakamoto et al., 1999).

*Labor requirement* – Similar to dwarf tomatoes, LTT does not require a mechanically raised platform to care for the plants, or a pipe rail trolley as plants are small-statured, thus labor requirement is lower than in high-wire (Itoh et al., 2020). However, as apical dominance is removed by pinching the main shoot in LTT, the requirement of side-shoots removal might be more frequent. It has been shown that in single-truss tomato systems, supporting and pruning, including pinching and side-shoots removal, represent major labor inputs (Giacomelli et al., 1994). Thus, our estimate of labor requirement in LTT used the labor requirements of dwarf tomato as a baseline, with additional labor for pruning and harvesting. Labor demand ranges from 0.15 – 0.20 min per plant for pruning and 0.11 min per plant for harvesting (Luxhoj and Giacomelli, 1990). With a planting density of 14.3 plants m^-2^ and four cultivation cycles each year, annual labor requirement is estimated at 5,400-5,900 h ha^-1^. At the projected maximum annual yield of 60 kg m^-^², this corresponds to approximately 0.54–0.59 min kg^-^¹.

## Discussion

The high-wire tomato production system has long dominated high-technology greenhouse tomato production. Over the years, yields and resource use efficiency have substantially increased by high-technology (e.g. precise climate control, and recirculating irrigation systems), but labor requirement remained largely the same. Although the demand for labor is relatively high, the high-wire system still has potential to develop in regions where labor is not a limiting factor. Therefore, its global dominance in tomato production is unlikely to be challenged in the near future. However, in some countries such as the Netherlands and Japan where labor is expensive and limited, the high labor requirements of high-wire systems have become a limitation, encouraging further automation.

Small-statured tomato plants, including dwarf tomato and limited-truss tomato (LTT), may facilitate more automated tomato cultivation by compact plants and simplified crop management. Compared to high-wire, crop management for dwarf tomatoes and LTT require less training or leaf removal, although side shoot removal could be challenging due to the bushy architecture of dwarf tomato and the absence of apical dominance caused by pinching in LTT. In a high-wire system, the growth of new leaves occurs simultaneously with fruit development for almost the entire cultivation cycle. In contrast, dwarf tomato and LTT allow greater temporal separation between canopy establishment and peak fruit filling. In dwarf tomato production, the canopy establishment occurs during the early phase of the cycle, while fruit growth becomes predominant later. In LTT, top pinching terminates further main-shoot leaf initiation, although the younger existing leaves continue to expand. This partial temporal separation between vegetative and generative growth opens new climate control possibilities, with conditions optimized more toward vegetative development during the early phase and toward fruit growth and quality during the later phase.

Currently, dwarf tomatoes are mostly marketed as potted plants for indoor gardening rather than grown commercially for tomato fruit production (Cruz and Gómez, 2022). The planting density is quite low, approximately 20 plants m^-2^ (Karpe et al., 2024). This low density is likely chosen because a corresponding attractive appearance is also a factor influencing consumer choice when buying potted plants. However, if dwarf tomatoes are grown for commercial fruit production in greenhouses or vertical farms, much higher and dynamic planting densities can be applied to improve yield and LUE (Karpe et al., 2024). Nevertheless, high-wire tomato remains a strong alternative to dwarf tomato due to the higher yield and proven crop management and fruit quality (size, tomato-on-the-vine, etc).

Several aspects need to be addressed before LTT can be widely adopted. Firstly, improving harvest index is important for LTT to increase LUE and yield, requiring further research on crop management practices such as the number of leaves retained and the number of trusses per plant. Retaining two leaves above the truss increased yield through bigger fruits compared to retaining no leaves (Logendra et al., 2001). When increasing the number of trusses from one to two, the yield significantly increased by 30% - 40%. However, adding a third truss did not result in a significantly higher yield than two trusses, mainly due to lower fruit set rates (Logendra et al., 2001). More than two trusses would also mitigate the advantage of separation of vegetative and generative growth. Furthermore, cultivars better suited for LTT need to be developed and selected. Since side shoots removal is one of the most time-consuming tasks in LTT, cultivars with reduced or no lateral shoot growth are needed. Notably, several recent studies on LTT have focused on Japanese cultivars, an evaluation of a broader range of genotypes is needed to better understand the adaptability to this system. Third, the high planting density and short crop cycle dramatically increase the number of seeds or seedlings required per unit area per year, making seed or seedling price an important determinant of production costs. Therefore, to become economically comparable to high-wire, seed or seedling costs in LTT need to be drastically reduced. Finally, existing relevant references are outdated (Luxhoj and Giacomelli, 1990), necessitating updated data to reflect current crop genetics and greenhouse management impacting labor demands.

In small-statured tomato cultivation systems, stage-specific climate control can be applied. Combined with sensing technologies, modelling, robotic harvesting etc., autonomous cultivation may be achievable. For example, one possible scenario involves the use of a movable gutter system to improve the working posture and optimize plants spacing (**Figure 1**). In this case, it is plants that are moved to workers or robots for management or harvesting (Giacomelli et al., 1994), rather than workers who move to the plants in conventional high-wire systems. Multiple compartments could be used to implement growth-stage-specific environmental conditions and crop management strategies. Sensors and cameras could be implemented to continuously monitor the environmental conditions and plant growth such as ground coverage percentage, pests and diseases, and fruit ripeness stage (Kaiser et al., 2024). With digital plant phenotyping, the yield and harvest time could be better predicted, and the robotic harvest could be developed within the movable gutter system. However, both too tall plants (which may need structural support) and too compact plants (which may restrict fruit accessibility) could reduce the ease of harvest automation, requiring further research on suitable plant architecture. This integration of cultivation system, facilities, sensor and camera data, and functional–structural plant modelling enables precise, efficient, and potentially autonomous management of the production process. Additionally, economic and energy feasibility should be evaluated before considering commercial implementation, as high annual yield does not necessarily result in better economic performance (Zhuang et al., 2024).

**Figure 1:**
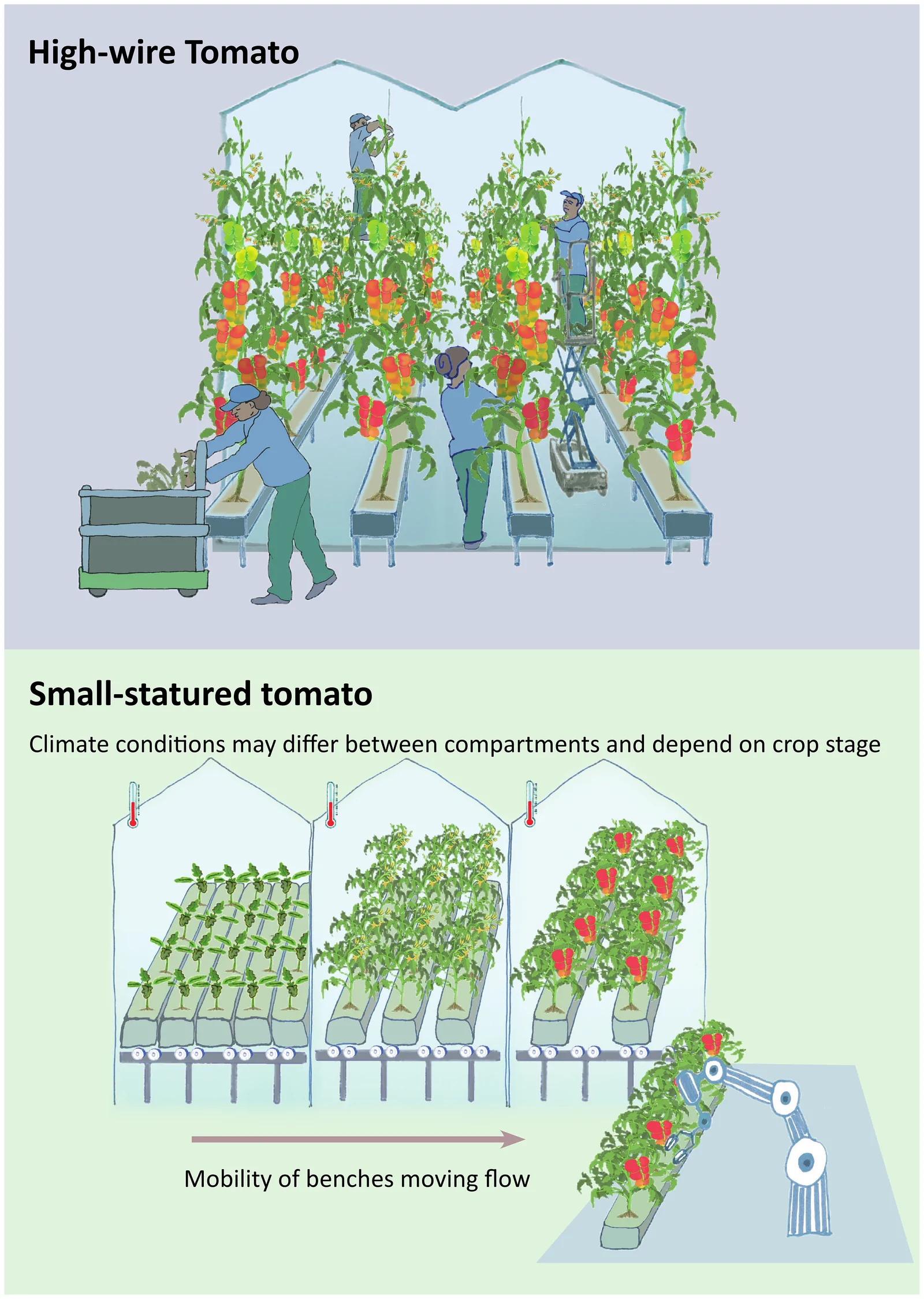
Schematic presentation of work patterns and automation levels in a current high-wire system (single climate zone) and a conceptual small-statured tomato system (multiple climate zones: climate depends on crop stage).

## Conclusion

Autonomous production will be a substantial part of future food production systems. The current standard production system for tomatoes, the high-wire system, limits automation. Small-statured plants, such as dwarf tomato and limited-truss tomato, may simplify crop architecture and enable more standardized crop handling and growth-stage-specific environmental control. We conclude that small-statured tomato systems represent a promising direction toward full automation, requiring multidisciplinary collaboration across plant physiological research, breeding, robotics and modeling. To make these systems commercial feasibility, further research is needed to improve yield, reduce labor costs, and optimize crop architecture.

## Data Availability

The original contributions presented in the study are included in the article/supplementary material, further inquiries can be directed to the corresponding author/s.
