# Supplementary material for: Small-statured tomato plants for automating greenhouse production: opportunities, constraints, and research priorities
Source: Front Plant Sci. 2026 Oct 28;17:1966159. doi: 10.3389/fpls.2026.1966159 (PMC13751512; doi:10.3389/fpls.2026.1966159)
Supplement: Supplementary file 1 [file Table1.pdf]

Supplementary Table S1. Summary of data sources and calculations used in Table 1.

System		Parameter	value	reference	reported /calculated	note, with reference to page (p) number in the reference
High-wire	large-fruited	planting density (stem m-2)	2.5-4	Heuvelink, E. (2018). Tomatoes	Reported	p 94, 2.5-3.75
		annual yield	111	KWIN 2026	Reported	p128, Tomato, vine (150-190g), w42, with lighting
		LUE（based on FW）	12	KWIN 2026	Calculated	annual yield divided by the sum of PAR light from solar radiation (p9), assuming a transmissivity of 70%, plus supplementary light (p128)
		LUE（based on DW）	0.5	• Schouten, I., Hawley, D., Olschowski, S., Ouzounis, T., Kerstens, T. N., Gianneas, T., Ludovico, J., Marcelis, L. F. M., & Heuvelink, E. (2024). Partially substituting top-light with intracanopy light increases yield more at higher LED light intensities. HortScience, 59(3), 421–428. https://doi.org/10.21273/HORTSCI17546-23<br>• Kusuma, P., Ouzounis, T., Hawley, D., Kerstens, T., Marcelis, L. F. M., & Heuvelink, E. (2023). On the pros and cons of red photons for greenhouse tomato production: increasing the percentage of red photons improves LED efficacy but plant responses are cultivar-specific. The Journal of Horticultural Science and Biotechnology, 98(4), 443–453.	Calculated	•LUE based on FW times a dry matter content of 3.8%
		HI	0.7	de Koning, A. N. M(1994). Development and dry matter distribution in glasshouse tomato: A quantitative approach. Wageningen University and Research.	Reported	p4, in a full-grown tomato crop the fruits are the main sinks as they receive about 70% of the total dry matter available for growth
		Labor requirement (h ha-1)	7500	KWIN 2026	Reported	p128, Tomato, vine (150-190g), w42, with lighting
		Labor requirement (min kg^{-1})	0.4	KWIN 2026	Calculated	60*(labor requirment/yield/10000)
		Soluble solids (°Brix)	4-6	Quinet, M., Angosto, T., Yuste-Lisbona, F. J., Blanchard-Gros, R., Bigot, S., Martinez, J.-P., & Lutts, S. (2019). Tomato fruit development and metabolism. *Frontiers in Plant Science* , *10* . https://doi.org/10.3389/fpls.2019.01554	Reported	p11, Numerous wild species differ from domesticated tomato cultivars and contain high TSS (Total Soluble Solid, a convenient proxy for sugar content) (more than 10% against 4–6% for S. lycopersicum)
	cherry	planting density	2.5-4	same to large-fruited		
		annual yield	34	KWIN 2026	Reported	p121, Tomato, cherry wine, w52
		LUE (based on FW)	4	KWIN 2026	Calculated	annual yield divided by the sum of PAR light from solar radiation (p9), assuming a transmissivity of 70%, plus supplementary light (p121)
		LUE (based on DW)	0.40	Hemming, S., de Zwart, F., Elings, A., Petropoulou, A., & Righini, I. (2020). Cherry Tomato Production in Intelligent Greenhouses—Sensors and AI for Control of Climate, Irrigation, Crop Yield, and Quality. Sensors, 20(22), 6430.	Calculated	LUE based on FW times a dry meter content 9%
		HI	0.7	same as large-fruited		
		Labor requirement (h ha-1)	17000	KWIN 2026	Reported	p121, Tomato, cherry wine, w52
		Labor requirement (min kg^{-1})	3.0	KWIN 2026	Calculated	60*(labor hours requirement/yield/10000)

		Soluble solids (°Brix)	6-14	• Bhandari, S. R., Chae, Y., & Lee, J. G. (2016). Assessment of phytochemicals, quality attributes, and antioxidant activities in commercial tomato cultivars. Horticultural Science and Technology, 34(5), 677–691. https://doi.org/10.12972/kjhst.20160071<br>• Ino, M., Ono, E., Shimizu, Y., & Omasa, K. (2023). Verification of commercial near-infrared spectroscopy measurement and fresh weight diversity modeling in brix% for small tomato fruits with various cultivars and growth conditions. Sensors, 23(12), 5460. https://doi.org/10.3390/s23125460	Reported	Bhandari's paper measured Brix of 13 cherry tomato cultivars, and values ranged from 6-9.<br>Ino's paper measured one high-sugar cultivar whose Brix was 14.
Dwarf		planting density (stem m-2)	20-100	Karpe, M., Marcelis, L. F. M., & Heuvelink, E. (2024). Dynamic plant spacing in tomato results in high yields while mitigating the reduction in fruit quality associated with high planting densities. Frontiers in Plant Science, 15. https://doi.org/10.3389/fpls.2024.1386950	Reported	p3, density was 100, 19, 54, and 41 plants/m2 averaged over 100 days from transplant to destructive harvest
		annual yield	30-45	Maree, S. C., Zhang, P., van Marrewijk, B. M., de Zwart, F., Bijlaard, M., & Hemming, S. (2025). Autonomous greenhouse cultivation of dwarf tomato: Performance evaluation of intelligent algorithms for multiple-sensor feedback. Sensors, 25(14), 4321.	Reported	p 14, the obtained yearly harvest was 45 kg/m2/year for IDEAS, 44 for MuGrow, 41 for Trigger, 35 for Reference, 32 for Tomatonuts, and 30 kg/m2/year for Agrifusion.
		LUE (based on FW)	7-11	Maree, S. C., Zhang, P., van Marrewijk, B. M., de Zwart, F., Bijlaard, M., & Hemming, S. (2025). Autonomous greenhouse cultivation of dwarf tomato: Performance evaluation of intelligent algorithms for multiple-sensor feedback. Sensors, 25(14), 4321. https://doi.org/10.3390/s25144321	Reported	p12
		LUE (based on DW)	0.7-1.1	Karpe, M., Marcelis, L. F. M., & Heuvelink, E. (2024). Dynamic plant spacing in tomato results in high yields while mitigating the reduction in fruit quality associated with high planting densities. Frontiers in Plant Science, 15. https://doi.org/10.3389/fpls.2024.1386950	Calculated	calculated according to Figure 3D and 3G
		HI	0.5-0.6	Karpe, M., Marcelis, L. F. M., & Heuvelink, E. (2024). Dynamic plant spacing in tomato results in high yields while mitigating the reduction in fruit quality associated with high planting densities. Frontiers in Plant Science, 15. https://doi.org/10.3389/fpls.2024.1386950	Reported	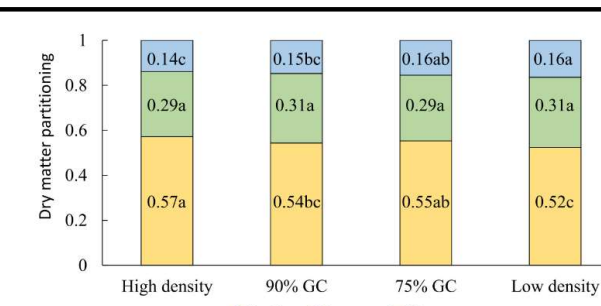
		Labor requirement (h ha-1)	4000	KWIN 2026	Reported	p204, Bromelia Vriesea on mobile benches
		Labor requirement (min kg^{-1})	0.5-0.8	KWIN 2026	Calculated	60*(labor hour requirment/yield/10000)

	Soluble solids (°Brix)	5-7	(Terada et al., 2023; Tharpe, 2023)(Karpe et al., 2024).	Reported	'Micro-Tom' reached 5.3–5.9 °Bx under different nutrition rates, while cultivars such as 'Micro Tom', 'Jochalos', and 'Venus' exhibited cultivar-dependent Brix values of 4.9–5.5 °Bx (Terada et al., 2023; Tharpe, 2023). In addition, fruit quality is also affected by planting density. For 'Plum Tomato Red', Brix decreased from 9.4 °Bx at constant low-density (19 plants m^{-2}) to 6.6 °Bx at constant high-density (100 plants m^{-2}), whereas dynamic plant spacing maintained values of 8.9 – 9.4 °Bx (Karpe et al., 2024).
	planting density (stem m-2)	10-14	• Jiang, C., Johkan, M., Hohjo, M., Tsukagoshi, S., Ebihara, M., Nakaminami, A., & Maruo, T. (2017). Responses of leaf photosynthesis, plant growth and fruit production to periodic alteration of plant density in winter produced single-truss tomatoes. The Horticulture Journal, 86(4), 511–518. https://doi.org/10.2503/hortj.OKD-060	Reported	treatment
	annual yield	24-60	(Itoh & Higashide, 2025; Jiang et al., 2017; Lu et al., 2012b, 2012a; Tewolde et al., 2016).	Calculated	3-15 kg/cycle, planting density 14.3, With a growth cycle of three months, excluding the four weeks of seedling production after sowing, four rounds can be achieved per year, resulting in an estimated maximum annual yield of 60 kg m-2.
	LUE (based on FW)	4-7	Itoh, M., Goto, C., Iwasaki, Y., Sugeno, W., Ahn, D.-H., & Higashide, T. (2020). Modeling and prediction of dry matter production by tomato plants in year-round production based on short-term, low-truss crop management. The Horticulture Journal, 89(4), 417–424. https://doi.org/10.2503/hortj.UTD-143	Calculated	fruit FW(kg/m2)*1000/cumulative incident photon(MJ/m2)/4.6
LTT	HI	0.4-0.6	• Jiang, C., Johkan, M., Hohjo, M., Tsukagoshi, S., Ebihara, M., Nakaminami, A., & Maruo, T. (2017). Responses of leaf photosynthesis, plant growth and fruit production to periodic alteration of plant density in winter produced single-truss tomatoes. The Horticulture Journal, 86(4), 511–518. https://doi.org/10.2503/hortj.OKD-060 • Sato, H., Yanagi, T., Hirai, H., Ueda, Y., & Oda, Y. (1994). Effects of shading on growth, fruit yield and dry matter partitioning of single truss tomato plants. Environment Control in Biology, 32(4), 231–237. https://doi.org/10.2525/ecb1963.32.231	Calculated	Calculated from fruit DW and total DW
	Labor requirement (h ha-1)	5400-5900	Luxhoj, J. T., & Giacomelli, G. A. (1990). Comparison of labour standards for a greenhouse tomato production system: A case study. *International Journal of Operations & Production Management* , *10* (3), 38–49. https://doi.org/10.1108/01443579010001591	Calculated	labour requirement of dwarf tomato plus 0.11 min per plant for harvesting plus 0.15 – 0.20 min per plant for purning
	Labor requirement (min kg^{-1})	0.54-0.59	Luxhoj, J. T., & Giacomelli, G. A. (1990). Comparison of labour standards for a greenhouse tomato production system: A case study. *International Journal of Operations & Production Management* , *10* (3), 38–49. https://doi.org/10.1108/01443579010001591	Calculated	60*(labor hours requirment/yield/10000)

	Soluble solids (°Brix)	Similar to high-wire, but can achieve higher Brix by imposing End-of-Production salinity stress
